# Dissociation between mental fatigue and motivational state during prolonged mental activity

**DOI:** 10.3389/fnbeh.2015.00176

**Published:** 2015-07-13

**Authors:** Mónika Gergelyfi, Benvenuto Jacob, Etienne Olivier, Alexandre Zénon

**Affiliations:** Institute of Neuroscience, Université Catholique de LouvainBrussels, Belgium

**Keywords:** mental fatigue, motivation, cognitive effort, EEG, ECG, pupillometry, eye blinks, skin conductance responses

## Abstract

Mental fatigue (MF) is commonly observed following prolonged cognitive activity and can have major repercussions on the daily life of patients as well as healthy individuals. Despite its important impact, the cognitive processes involved in MF remain largely unknown. An influential hypothesis states that MF does not arise from a disruption of overused neural processes but, rather, is caused by a progressive decrease in motivation-related task engagement. Here, to test this hypothesis, we measured various neural, autonomic, psychometric and behavioral signatures of MF and motivation (EEG, ECG, pupil size, eye blinks, Skin conductance responses (SCRs), questionnaires and performance in a working memory (WM) task) in healthy volunteers, while MF was induced by Sudoku tasks performed for 120 min. Moreover extrinsic motivation was manipulated by using different levels of monetary reward. We found that, during the course of the experiment, the participants’ subjective feeling of fatigue increased and their performance worsened while their blink rate and heart rate variability (HRV) increased. Conversely, reward-induced EEG, pupillometric and skin conductance signal changes, regarded as indicators of task engagement, remained constant during the experiment, and failed to correlate with the indices of MF. In addition, MF did not affect a simple reaction time task, despite the strong influence of extrinsic motivation on this task. Finally, alterations of the motivational state through monetary incentives failed to compensate the effects of MF. These findings indicate that MF in healthy subjects is not caused by an alteration of task engagement but is likely to be the consequence of a decrease in the efficiency, or availability, of cognitive resources.

## Introduction

Mental fatigue (MF) is a recurring problem in the daily life of many people and remains a challenging symptom for clinicians (Walker et al., 1993; Pawlikowska et al., 1994). In healthy subjects it can be the consequence of prolonged and intense cognitive activity (van der Linden et al., 2003), while in patients, it can become a permanent condition (Millikin et al., 2003). MF consists primarily in the subjective feeling of a deteriorated ability to engage in mental activities, but it can also be objectively measurable in terms of performance decrements (Schwid et al., 2003; Lorist et al., 2009). MF is distinct, albeit closely related to the concept of vigilance (Thiffault and Bergeron, 2003), which refers more specifically to the capacity to sustain attention over time. Vigilance is typically assessed by measuring the speed and variability of responses during simple detection tasks, such as the psychomotor vigilance task (PVT), and/or by evaluating electrophysiological changes in the EEG theta band (Paus et al., 1997). Vigilance typically decreases following sleep deprivation (Dorrian et al., 2005) while, in the absence of sleep deprivation, drops in vigilance have been attributed to “boredom”, i.e., the incapacity to maintain sustained attention during simple unstimulating tasks (Frankmann and Adams, 1962; Mackworth, 1968), even though some studies have questioned this view (Smit et al., 2004a,b; Gunzelmann et al., 2011).

The cognitive mechanisms at the origin of MF remain poorly understood. In particular, it is still unclear whether the decreased performance associated with MF is caused by a progressive deterioration of the cognitive resources (e.g., attention, memory) or by an inadequate recruitment of unaltered cognitive processes, caused by a loss of motivation. To date, despite the importance of this question (Chaudhuri and Behan, 2000; Hockey, 2011; Kurzban et al., 2013; Botvinick and Braver, 2014) only few studies have looked at the relation between MF and motivation. Chaudhuri and Behan (2000) have proposed that MF could, at least partly, result from a loss of motivation to engage in self-initiated tasks and that pathological fatigue would be the consequence of an alteration of the motivational brain circuits, including the basal ganglia. Tops et al. (2004) suggested that MF could be viewed as an effort/reward imbalance: when the effort is proportionally larger than the associated reward, the motivation to engage in the task decreases and MF appears. Along the same lines, Hockey (2011) and others (Kurzban et al., 2013; Botvinick and Braver, 2014) have argued that fatigue consists in a control mechanism that drives individuals away from prolonged tasks and towards newer, potentially more rewarding activities.

However, previous studies have found that monetary incentives provided after fatigue inducement failed to recover the pre-fatigue level of performance, suggesting that MF might not depend only on a cost/benefit imbalance (Boksem et al., 2006; Lorist et al., 2009). In a recent study representative of this approach, Hopstaken and colleagues measured various psychophysiological variables during a fatiguing task (Hopstaken et al., 2014). They found that following fatigue, increasing extrinsic motivation recovers pre-fatigue level of performance, and argued that this provides evidence in favor of a fatigue-induced task disengagement from the task. However, without assessing the effect of extrinsic motivation on performance before fatigue inducement, it is impossible to interpret this finding, because performance is evaluated in two different motivational states: a low motivational state and a high motivational state before and after fatigue inducement, respectively. This confound also applies to previous studies based on the same principle (Boksem et al., 2006; Lorist et al., 2009). In addition, the pupillometric findings from Hopstaken et al. (2014), showing that pupil baseline diameter decreased with time-on-task, suggest, in contrast to the behavioral results, that task engagement increased over time (Gilzenrat et al., 2010), arguing against a task disengagement account for MF.

In addition to its subjective and behavioral effects, fatigue is also known to impact psychophysiological and neurophysiological variables. In a range of studies using cognitive tasks (e.g., the Stroop task or a simulated driving/flight task) over a prolonged period of time, EEG correlates of fatigue have been investigated. The most consistent changes observed with time-on-task have been an increase in the low frequency bands (delta: 0–4 Hz; theta: 4–8 Hz and alpha: 8–12 Hz) together with an decrease in the beta frequency bands (12–20 Hz; Lal and Craig, 2002; Boksem et al., 2005; Barwick et al., 2012; Borghini et al., 2012a,b; Craig et al., 2012; Zhao et al., 2012; Wascher et al., 2014), and an increase in the P300 latency (Kaseda et al., 1998; Kato et al., 2009). Another technique used to track MF is the ECG: the heart rate decreases while the heart rate variability (HRV) increases with MF (Egelund, 1982; Mascord and Heath, 1992). Fairclough et al. (2005), for instance, found that the power of middle-frequency component of the HRV (0.1 Hz sinus arrhythmia) increased while the participants performed the Multi-attribute Task Battery over a period of 64 min. In addition to its relation to MF, the heart rate and HRV also index task difficulty, such that higher heart rate and lower HRV are associated with larger effort mobilization (O’Hanlon, 1972; Mulder, 1986; Veltman and Gaillard, 1996; Schellekens et al., 2000). This opposite pattern of ECG correlates between MF and mental effort suggests that MF could indeed be associated to lower effort investment, in accordance with the motivational account of MF. Finally, in addition to these electrophysiological correlates, eye blink parameters are also known to vary with time-on-task (Stern et al., 1994; Van Orden et al., 2001). The average blink duration, and the proportion of long blinks, have been associated to subjective drowsiness (i.e., the propensity to fall asleep, Caffier et al., 2003), vigilance drops (McIntire et al., 2014) or performance drops during prolonged tasks following sleep deprivation (Morris and Miller, 1996). In contrast, blink frequency may be more related to MF *per se* (Martins and Carvalho, 2015).

In the present study, we aimed to investigate whether MF is caused by a progressive disengagement from the task. We evaluated MF throughout the execution of a Sudoku task by using subjective ratings, objective behavioral performance, neural and autonomic variables known to covary with MF: EEG, ECG and blink rate. We then evaluated intrinsic motivation and measured the effect of the manipulation of extrinsic motivation on behavior and on a series of physiological factors known to be sensitive to mental effort, and thus providing an indirect measure of task engagement: pupil diameter (Beatty, 1982), EEG (Venables and Fairclough, 2009) and skin conductance response (SCR; Kahneman, 1973; Pessiglione et al., 2007). Importantly, we assessed the effect of extrinsic motivation at different time points during the experiment, allowing motivational effects on behavior to be compared before and after fatigue inducement. Finally, in order to evaluate task-unspecific fatigue effects and to get rid of the boredom confound caused by the repeated execution of the same task for prolonged periods of time (Hockey, 2013), we used different tasks to induce (Sudoku task) and measure [working memory (WM) task] fatigue.

The hypothesis that MF would be caused by a disengagement from the task led us to make the four following predictions: (1) task engagement should decrease over time; (2) this decrease should correlate with subjective and/or objective assessments of MF; (3) reward manipulation should at least partly alleviate the detrimental effect of MF; and (4) MF should influence all tasks that are sensitive to motivational factors.

## Materials and Methods

### Participants

Eighteen healthy subjects (eight females) participated in this study (age = 25.72 ± 3.54, mean ± SD). The participants were recruited in accordance with the following criteria: they had to be performing at least one Sudoku grid per month, to be aged between 20–35 years old, had to be right-handed, have normal or corrected-to-normal visual acuity, not to be under medical treatment and not to be a smoker. Subjects provided written informed consent prior to the experiment and receive financial compensation for their participation (~60–80 euros). All the participants were naive regarding the aim of the study. The study was approved by the local ethics committee (Comité d’ Ethique, hospital-facultaire de l’UCL).

### Tasks

In order to keep the subjects intrinsically motivated during the experiment we used the indirect method to assess the behavioral effects of MF (Ackerman, 2011). The indirect method consists in using different tasks to induce and to evaluate fatigue. MF was induced by performing a computerized version of the popular Sudoku game. The behavioral effect of MF was measured by using a WM task in which extrinsic motivation was manipulated by means of different levels of reward. The effect of this reward manipulation was controlled with a simple RT (SiRT) task.

#### Mental Fatigue Inducement: Sudoku Puzzle

**Figure 1:**
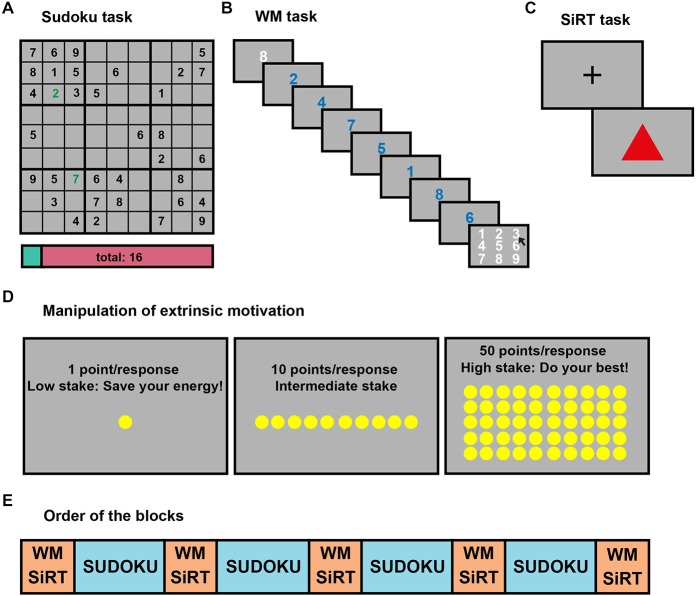
**Experimental tasks and design. (A)** An example of a Sudoku puzzle used to induce mental fatigue (MF). Incorrect responses were highlighted by a red colored number and signaled by an auditory signal, which faded away after 1.5 s, while correct responses were displayed in green and triggered a sound sample corresponding to the Japanese word for that number. The points already earned were displayed on the score bar at the bottom of the screen, and were illustrated graphically by the proportion of the cyan color on that bar. Whenever a grid was fully completed, a brief music sample was played and a short fireworks animation was shown on the screen. **(B)** In the MF evaluating working memory (WM) task, the length of the number series (called condition 8) was first presented in white for 1500 ms, followed by the numerals composing the series (2, 4, 7, 5, 1, 8, 6), in blue. Each numeral was displayed for 400 ms. The missing element (3) of the number series had to be selected with the computer mouse from a numeric keypad shown on the screen. **(C)** In the control SiRT task, the subject had to press the left mouse button as fast as possible when a red triangle appeared on the screen. A fixation cross (+) was displayed between these stimulus presentations for a duration varying, according to a geometric distribution, from 500 to 3000 ms. The RTs of the subjects were summed up after each trial and the task ended when this sum reached a total of 6000 ms. **(D)** The reward condition (1, 10 or 50 points) and recommended strategy was instructed at the beginning of each block. When the reward was 1 point per correct response, we proposed them to “save their energy”, and they were advised “to do their best” when the reward was 50 points. **(E)** Order of the MF evaluation and inducement blocks. Each 30 min-long MF inducement blocks (four blocks in blue) consisted of Sudoku puzzles, while each 14 min-long MF evaluation blocks (five blocks in orange) included six sub-blocks of SiRT and six sub-blocks of WM tasks. These sub-blocks were randomly interleaved, and each block was repeated twice as a function of reward condition (1, 10, 50 points).

The objective of the Sudoku puzzle is to fill the cells of a 9 × 9 grid, further divided into 3 × 3 subgrids, with digits from 1 to 9, every digit occurring only once in a row, a column and a subgrid of the puzzle (see Figure 1A). Subjects entered their response by clicking the left mouse button on a Sudoku cell; this made a numerical keypad to pop up, allowing them to pick a number to fill the cell. Feedback was given on each cell, thus the subjects could use a strategy consisting in picking randomly the numbers until the right one comes up. In order to prevent this strategy, we applied a scoring system with +8 points per correct response and −20 points per wrong response. At the beginning of each Sudoku task, the average number of empty cells was 47 ± 11, and thus, the maximum amount of points that the subjects could win for a single grid was around 375.

Sudoku grids were collected from a website^1^, categorizing the grids into four difficulty levels: easy, medium, hard and absurd. In addition, within each of these categories, grids are assigned a given number of stars (from 1 to 6) specifying more precisely their difficulty level. This particular scoring method has been validated in a previous computational study, using Sudoku grids from the same website (Ercsey-Ravasz and Toroczkai, 2012).

We pretested the Sudoku grids having different difficulty levels in a pilot study performed on a group of healthy subjects (*n* = 8, age 20–35 years). This allowed us to confirm that higher difficulty levels led to higher median reaction time (RT). It also showed that, given the variability in performance between the subjects, the difficulty level had to be adjusted on a subject-by-subject basis. Based on these findings, we established the following rule to choose the appropriate difficulty level for each subject participating in the main experiment. During a training session, the median of the RTs was computed for each grid performed. For median RT under 5 s or above 10 s, a more difficult or an easier Sudoku task was given to the subject, respectively. If the median RT was under 5 s but the number of errors was above 5 per grid, the difficulty level was maintained constant. It could occur that the easiest Sudoku task was too difficult for a subject; in this case some of the cells of the Sudoku grid were prefilled in order to make it easier. The procedure was stopped, and the level of each subject determined, whenever a grid was completed with a median RT between 5 and 10 s, and less than 5 errors per grid. This procedure resulted in 13 subjects undergoing easy Sudoku grids (mean ± SD “stars” number: 2.7 ± 0.4) and five subjects performing Sudoku grids of medium difficulty (“stars” number: 2.8 ± 0.6).

During the experimental session, the Sudoku task was performed for 2 h (four blocks of 30 min), intermingled with five blocks of MF evaluation (see below for further details).

#### Mental Fatigue Measurement: Working Memory (WM) Task

The cognitive effect of MF was measured by means of a WM task in which extrinsic motivation was manipulated by using three different levels of reward: 1, 10 and 50 points. In order to increase the difficulty of the task, two different conditions were randomly interleaved during each block of 14 trials. In a first condition called “8”, seven numerals ranging between 1 and 8 were presented in a random order (e.g., 2, 4, 7, 5, 1, 8, 6) and the task consisted of reporting the missing number (“target” number) in the series (3 in the above example, see Figure 1B). In the “9” condition, eight numerals between 1 and 9 were presented, and the task was the same as above. The subjects did not receive any feedback about their performance.

At the beginning of each 14-trial block of the WM task, the participants were informed about the actual amount of reward (1, 10 or 50 points) that they could gain for each correct response in the upcoming block. In addition, for the 1 and 50 points conditions, a sentence displayed on the screen provided a recommendation about the strategy the subjects should adopt to maximize their earnings (see Figure 1D). Following the display of the reward condition, the participants were allowed to trigger the task whenever they wished by pressing a key on the keyboard. The “decision time” that was spent between the display and task onset was measured. At the end of 14 trials, the final score was shown on the screen for 2000 ms. Subjects were aware that these points were converted into actual money at the end of the experiment.

#### Control of Extrinsic Motivation: Simple Reaction Time (SiRT) Task

To control the effect of the reward manipulation on extrinsic motivation, six blocks of SiRT task were also performed between each Sudoku block, randomly interleaved with the WM task. In this control task, participants had to press the left mouse button as fast as possible every time a red triangle appeared on the screen (see Figure 1C). Given that the sum of response times was fixed during a block, the faster the participants responded, the more trials they could perform and the more points they could gain.

The Sudoku, WM and SiRT tasks were implemented in Matlab 7.5 (The MathWorks, Natick, MA, USA) and were displayed by means of the psychophysics toolbox (Brainard, 1997) and an in-house graphics toolbox (CosyGraphics).

### Physiological Measurements

EEG signals were acquired by means of the ASA-lab recording system (ANT Inc., Netherlands) using 32 Ag-AgCI electrodes placed on the scalp based on the international 10/20 system (Waveguard32 cap, Cephalon A/S, Denmark). Recordings were made in an electrically shielded room. Horizontal and vertical eye movements (saccades) and blinks were also recorded using two additional surface electrodes placed close to the outer canthus of the right eye and on the upper-left side of the right eye. Two additional electrodes were attached to the left and right forearms in order to monitor the electrocardiographic (ECG) signal. Electrode impedances were kept below 25 kΩ. The signals were amplified and digitized using a sampling rate of 500 Hz.

An EyeLink 1000 eye-tracker (SR Research Ltd., Kanata, ON, Canada) monitored eye movements, blinks and pupil diameter at a sampling frequency of 500 Hz.

SCRs were recorded by means of a CED 2502 skin conductance unit (Cambridge Electronic Design, UK) and sampled at 100 Hz.

### Subjective Measures

Finally, a modified version of the Multidimensional Fatigue Inventory (MFI; Gentile et al., 2003) and the Post-experimental Intrinsic Motivation Inventory (IMI)^2^, were used to evaluate the participants’ fatigue and intrinsic motivational state at the beginning and at the end of the experiment. The MFI contains 20 items classified into four dimensions: general fatigue, MF, reduced activities and motivation. The statements have to be rated on a 5-point Likert scale (from “Yes, that is true” to “No, that is not true”) representing the subject’s current feeling. Low MFI scores reflect a higher degree of fatigue. From the 45 items included in the Post-experimental IMI, we used only those included in the three subscales we deemed relevant to assess intrinsic motivation in our task, namely interest/enjoyment, perceived competence and effort/importance. In this questionnaire, participants have to rate each item on a 7-point Likert scale (from “Not at all true to “Very true”). The higher the score on a subscale, the more the given trait is represented.

Additionally, the State-Trait Anxiety Inventory (STAI; Spielberger, 1983) and the Beck Depression Inventory (BDI; Beck et al., 1961) were used to assess trait anxiety and depression. The STAI is a 40-item inventory divided into two subscales. We used only the second 20-item subscale in this study in order to evaluate the subject’s anxiety traits. A score between 1 and 4 (from “Almost never” to “Almost Always”) has to be provided for each statement. Higher scores suggest more severe degree of anxiety. The 21-item BDI has different groups of statements. A score from 14 to 19 indicates a mildly depressed state while a score over 20 is indicative of clinical depression.

### Design and Experimental Procedure

The participants (*n* = 18) had to attend two sessions (training and experiment). The aim of the training session was to tune the difficulty level of the Sudoku task to the subject’s ability (see above) and to practice the WM task in order to decrease the influence of training during the main experiment. This training session was always conducted in the morning (between 10:00 and 12:00 AM) and took about 90 min. At the beginning of this session the subjects received instructions related to the tasks (Sudoku task, SiRT task and WM task) and were informed about the experimental procedure. Then, the subjects underwent the Sudoku difficulty tuning procedure described above. Thereafter, they practiced six blocks of the WM task. Subjects received auditory feedback about their performance only in the three first blocks of this task. Participants also performed the SiRT task once.

At the end of this training session, every subject received five Sudoku grids adapted to his/her ability and two inventories (BDI and STAI) to be filled at home. The Sudoku tasks and the inventories had to be completed and sent back before the second, experimental session. At least 1-day break was imposed between the two sessions.

The experimental session was always run in the afternoon or early in the evening (between 2:00 and 8:00 PM) and lasted between 4 and 5 h. Participants first filled out the MFI and IMI questionnaires. Then they seated in front of the computer for the remaining of the experimental session, at a distance of 0.80 m from the PC monitor in a dimly illuminated, electrically shielded and sound-attenuated room. The EEG cap was then placed, the impedance of the electrodes adjusted and then EyeLink camera and skin conductance unit was set up. Immediately before the experiment, written instructions were given to the subjects. The 190 min-long experiment was then started, in which four 30-min blocks of Sudoku were performed, intermingled with five blocks of MF evaluation (see Figure 1E). In each Sudoku block, different grids were provided to the subject until the 30-min time limit was reached (mean number of grids performed: 3 ± 1). No break was allowed between the nine blocks (5 MF evaluation + 4 Sudoku blocks). At the end of the experiment, the subjects had to complete the same inventories as before the experiment. The order of the statements in these inventories was pseudo-randomized. Finally, the points collected during the Sudoku, the WM and the SiRT tasks were converted into euros and added together to determine compensation that the participants received at the end of the experiment.

### Data and Statistical Analyses

#### Behavioral Data

Most statistical analyses were performed with Matlab 7.5 (The MathWorks, Natick, MA, USA) and consisted in *t*-tests, two-way repeated-measures ANOVAs with BLOCK (1–5) and REWARD (1, 10 or 50) as independent variables and Pearson correlations whenever the normality of the data allowed us to use this test. Normality was ensured by examining the residuals by means of a Q-Q plot. In particular, the residuals from the accuracy data, which typically exhibit a skewed distribution, were in our case satisfactorily normal, allowing us to use simple parametric tests. When necessary, log-transformation (Keene, 1995) was used on non-normally distributed data. In some instances reported in the results section, when transformations failed to normalize the data, Spearman correlations were used. Pairwise *post hoc* comparisons were Bonferroni corrected. If Bonferroni adjusted *post hoc* tests failed to reach significance despite significant main effects, ANCOVAs were used to evaluate the continuous effect of BLOCK and REWARD.

#### Physiological Data

In order to evaluate whether a given physiological variable could be regarded as a marker of motivation, we followed the same rationale as in previous studies (Locke and Braver, 2008; Schmidt et al., 2012), namely we considered that: (1) it should correlate with the reward value in the WM task; and (2) the reward-induced signal changes should correlate with the reward-induced changes in behavior. These correlations were performed by means of ANCOVAs including the physiological variable as dependent variable, the behavioral performance as continuous independent variable and the subject as random factor. In order to ensure that the correlations reflected only the reward-induced changes that were common in the physiological marker and the behavioral performance, the effect of the block was removed from both variables by subtracting the blockwise average from the data prior to running the ANCOVA. Conversely, to assess the correlation between the effect of blocks (i.e., the fatigue effect) on the behavior and on the physiological markers, the reward-induced changes were removed by subtracting the reward-wise averages from the data.

##### EEG data

We focused our analysis of the EEG data gathered solely during the WM task. Two subjects were discarded from this analysis because of a technical issue. The EEG processing steps were performed by means of Letswave 5^3^ and Matlab 8.0 (The MathWorks, Natick, MA, USA). Continuous EEG recordings were average-referenced offline. Ninety-second long EEG epochs were aligned on the onset of each WM task and transformed into the frequency domain using a fast Fourier transform. The frequency spectrum was then cropped between 0 and 150 Hz with a 0.5 Hz resolution, log-transformed and z-scored in order to ensure the normality of the distribution. Average EEG spectral power was computed for each subject and condition in six frequency bands: delta (0–4 Hz), theta (4–7 Hz), alpha (8–12 Hz), beta (15–30 Hz), low gamma (30–60 Hz) and high gamma (60–140 Hz). The effects of reward and block order on EEG activity were investigated separately by means of regressions on dependent samples with cluster-based permutation tests, correcting for multiple comparisons (Oostenveld et al., 2011).

##### ECG data

**Figure 2:**
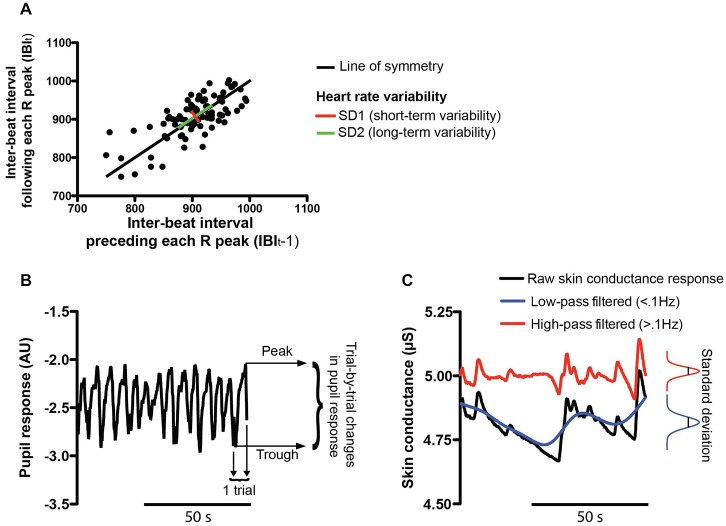
**Physiological measures. (A)** The heart rate variability (HRV) was assessed by Poincaré plot analysis which represents each inter-beat interval (IBI) against the IBI that precedes it (IBI_t_ vs. IBI_t−1_). This analysis results in two statistics: the short-term HRV (SD1 in red) is the standard deviation perpendicular to the line of symmetry while the long-term HRV (SD2 in green) is computed along the line of symmetry of the plot. **(B)** Example pupil size recording during the WM task. The pupil response was computed as the trialwise peak-to-peak difference. Lower and upper peaks of the pupil response in an example trial (trial n°14) are represented by black arrows. **(C)** Example skin conductance signal obtained during the WM task. The standard deviation of the amplitude of SCRs was computed for different frequency bands. The raw SCRs are shown in black while SCRs filtered in the low (<0.1 Hz) or high frequency band (>0.1 Hz) are represented in blue and red, respectively.

The same subjects as for the EEG analyses were excluded. ECG signals were preprocessed similarly to the EEG signals. The segmentation of the ECG signals also resulted in 30 90-s long epochs for every subject. Thereafter, a finite impulse response band-pass filter with cut-off frequencies 10–20 Hz was used. The R peaks of the ECG signals were detected offline and the inter-beat interval (IBI) between the consecutive R deflections was computed. To assess the HRV, we used the non-linear Poincaré plot analysis (Piskorski and Guzik, 2007), which consists in representing each IBI against the IBI that precedes it (IBI_t_ vs. IBI_t−1_) (see Figure 2A).

##### Pupil diameter and eye blink parameters

In the WM task, data were also missing for two subjects (different from the ones excluded from the EEG analysis) because their unconstrained head movements resulted in the loss of the pupil signal and thus they had to be excluded from additional analyses. Pupil response was computed as the difference between the peak amplitude of the pupil during each trial and its baseline at trial onset (Zénon et al., 2014; see Figure 2B). Then, the median of these trial-by-trial changes in pupil size was calculated blockwise. Blink rate as a measure of MF was also computed for every subject and log-transformed. Furthermore, the median of the blink durations (log-transformed) and the proportion of long blink durations (proportion of blinks lasting longer than 300 ms, Caffier et al., 2003) were also computed blockwise.

##### Skin conductance response (SCR)

Eight consecutive subjects had to be excluded from the analysis due to a technical problem, which resulted in the data not being properly recorded. The low-pass and high-pass filtered SCR responses were analyzed separately (cutoff frequency 0.1 Hz, see Figure 2C). In each frequency range, the standard deviation of the amplitude of the SCRs was computed for every condition across subjects.

## Results

### Evidence for MF Emergence During the Experiment

**Figure 3:**
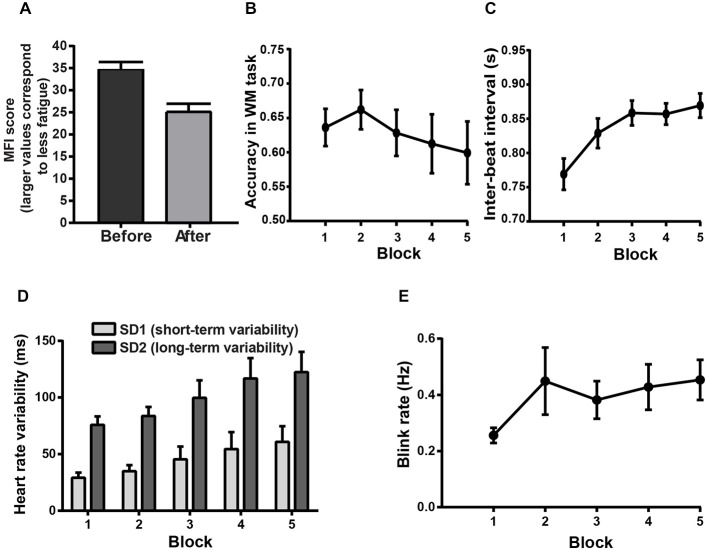
**Psychometric, behavioral and physiological evidence for MF.** Error bars represent standard errors of the mean. **(A)** Mean score on the general fatigue subscale of the Multidimensional Fatigue Inventory (MFI) before and after the experiment. Following the experiment, smaller average MFI indicates a higher level of subjective fatigue. **(B–E)** Block-related behavioral and physiological changes indicative of MF in the WM task. **(B)** Mean accuracy in the WM task across block repetition. **(C)** Mean values for IBI in the WM task across block repetition. **(D)** Short- and long-term HRV in the WM task as a function of the block order. **(E)** Average blink rate in the WM task as a function of block order.

The Sudoku task was performed with high accuracy (95.84% of correct responses) with a median RT ranging between 3.16 and 8.53 s per Sudoku cell; this task was successful in inducing MF, as shown in the following psychometric, behavioral and physiological analyses. Scores on the general fatigue subscale of the MFI showed that the subjects were significantly more fatigued after the experiment than before (paired samples *t*-tests: *t*_(17)_ = 5.56, *p* < 0.0001, see Figure 3A). In the WM task, two-way RM ANOVA on accuracy revealed a significant main effect of BLOCK (*F*_(4,68)_ = 2.52, *p* < 0.05, see Figure 3B). An ANCOVA including the BLOCK condition as a continuous variable was performed to investigate the nature of this effect. The best result was obtained when using the square of the block number (quadratic BLOCK effect: *F*_(1,485)_ = 4.15, *p* = 0.0436, linear BLOCK effect: *F*_(1,485)_ = 4.01, *p* = 0.047), showing that the subjects’ performance, following an initial improvement, got gradually worse over time (see Figure 3B).

We investigated whether this drop in performance correlated with the increased level of subjective fatigue. Based on earlier studies (Krupp and Elkins, 2000; Bryant et al., 2004), we did not expect to see any correlation between the subjective and objective indices of fatigue. The objective fatigue index was computed by taking the slope of the regression line between the accuracy in the WM task and block order, starting from block 2 to avoid the initial effect of training. We found a non-significant trend toward a positive correlation (Pearson correlation: *r* = 0.45, *p* = 0.059), i.e., subjects with a large increase in subjective fatigue paradoxically tended to maintain a higher level of performance in the WM task across block repetitions.

In addition to subjective and behavioral measures, physiological measures also provided evidence for the emergence of MF. A physiological variable was regarded as an index of MF when it was found to vary across block repetition in such way that correlated with the time-induced changes in behavior.

Cluster-based permutation tests were performed on the EEG electrodes and frequency bands to assess the effect of block on the EEG signal during the WM task (see “EEG Data” Section). In the high gamma band, a marginally significant BLOCK effect (*p* following cluster-based correction = 0.0842) was revealed in the left frontal region (F7, FC5) showing that the amplitude tended to increase across block repetitions, possibly corresponding to an electrophysiological signature of MF. The BLOCK effect on the other frequency bands was not significant (*p* > 0.1).

The heart rate analysis unveiled a main effect of BLOCK (two-way RM ANOVA: *F*_(4,60)_ = 27.33, *p* < 0.0001, see Figure 3C) on the IBI. This revealed that heart rate decreased across block repetition, presumably reflecting an increased level of fatigue (*post hoc* tests: all comparisons with block 1, *p* < 0.0001; block 3 and 5 vs. block 2, *p* < 0.001). The non-linear Poincaré plot analysis used to assess the HRV (see Figures 2A) allowed us to reveal a main effect of BLOCK (two-way RM ANOVA: *F*_(4,60)_ = 11.84, *p* < 0.0001, see Figure 3D) on SD1. This shows that the short-term HRV (SD1) also increased over time (*post hoc* tests: HRV was higher in the 3rd, 4th and 5th blocks compared to the 1st block, and in the 4th and 5th block compared to the 2nd and 3rd block, all *p* < 0.05). The long-term HRV (SD2) also increased across block repetition (two-way RM ANOVA: *F*_(4,60)_ = 13.32, *p* < 0.0001, see Figure 3D). *Post hoc* tests showed that SD2 increased significantly in the 3rd, 4th and 5th block compared to the 1st and 2nd blocks. Significant increase of SD2 was also observed in the 4th and 5th blocks compared to the 3rd block (all *p* < 0.05).

Furthermore, for the blink rate, a significant main effect of BLOCK order was found (two-way RM ANOVA: *F*_(4,60)_ = 3.77, *p* < 0.01, see Figure 3E) which is also known to be sensitive to fatigue (Martins and Carvalho, 2015). The blink rate increased as a function of block order, reflecting an increasing level of fatigue (*post hoc* tests: 4th and 5th block higher than the 1st block, all *p* < 0.05). The main effect of BLOCK order was also significant for the blink duration (two-way RM ANOVA: *F*_(4,60)_ = 2.56; *p* = 0.0476), however the *post hoc* tests with Bonferroni correction failed to reveal any significant pairwise comparison (all *p* > 0.1). In addition, an ANCOVA including the block condition as a continuous variable failed to show any significant BLOCK effect (quadratic BLOCK effect: *F*_(1,191)_ = 0.44; *p* = 0.5095; linear BLOCK effect: *F*_(1,191)_ = 1.05; *p* = 0.3079). Two-way RM ANOVA on the long blink durations showed only a marginally significant BLOCK effect (*F*_(4,60)_ = 2.12; *p* = 0.0889). The lack of significant block effect on these eye blink parameters was not unexpected, since eye blinks appear related more consistently to drowsiness and drops of vigilance rather than MF (Morris and Miller, 1996; Caffier et al., 2003; McIntire et al., 2014).

The block-related changes in the long-term HRV (SD2) and blink rate were negatively correlated with the block effect on WM task performance (ANCOVAs, SD2: *F*_(1,223)_ = 6.94, *p* = 0.009; blink rate: *F*_(1,223)_ = 6.04, *p* = 0.0148), confirming that these changes on SD2 and blink rate could be regarded as indices of MF. In contrast, blink duration correlated positively with WM task performance (*F*_(1,223)_ = 9.7, *p* = 0.0021). This appeared to be caused by the initial improvement in the WM task accuracy between the 1st and 2nd block, accompanied by a concurrent increase in blink duration. To account for this potential confound, Pearson correlation analyses were performed. We first computed the slope of the regression of blink duration and accuracy as a function of the block order, starting from the 2nd block. Then we computed the correlation between these values. This analysis revealed a non-significant negative correlation between the subject-by-subject slopes of the block effect on blink duration and the subject-by-subject slopes of the block effect on WM task accuracy (Pearson correlation: *r* = −0.0291; *p* = 0.9148), confirming that the changes in blink duration failed to track the changes in MF. The time-related changes in IBI and short-term HRV (SD1) also failed to show any significant correlation with the time-related changes in the WM task results (ANCOVAs, IBI: *F*_(1,223)_ = 2.2, *p* = 0.1399; SD1: *F*_(1,223)_ = 1.19, *p* = 0.2764), and thus these latter measures were not considered as markers of MF in the current study.

Pearson and Spearman correlations were performed between the change in the subjective feeling of fatigue (general fatigue subscale of the MFI) and subject-by-subject slopes of the block effect on each of the physiological measures of MF (IBI, SD1, SD2, blink rate, blink duration). None of these correlations were significant (all *p* > 0.1).

Low scores in the second subscale of the STAI (37.28 ± 8.27, mean ± SD) and in the BDI (5.39 ± 4.35, mean ± SD) showed that the subjects did not suffer from any anxious or depressive disorders when they participated in the experiment. Furthermore, these scores did not significantly correlate with the subjective feeling of fatigue (Spearman correlations, STAI: *r* = −0.2673, *p* = 0.2837; BDI: *r* = −0.2917; *p* = 0.2402), indicating that anxiety and depression did not play any role in the development of subjective fatigue.

### Evidence for Motivation Manipulation Efficacy

The efficacy of reward manipulation (1, 10 or 50 points) on behavioral performance was studied both in the SiRT and fatigue-evaluating WM task. In addition, we looked at the effect of the reward on the physiological variables, which were considered as valid markers of motivation whenever they varied in proportion to the reward value and correlated with the reward-induced behavioral changes.

**Figure 4:**
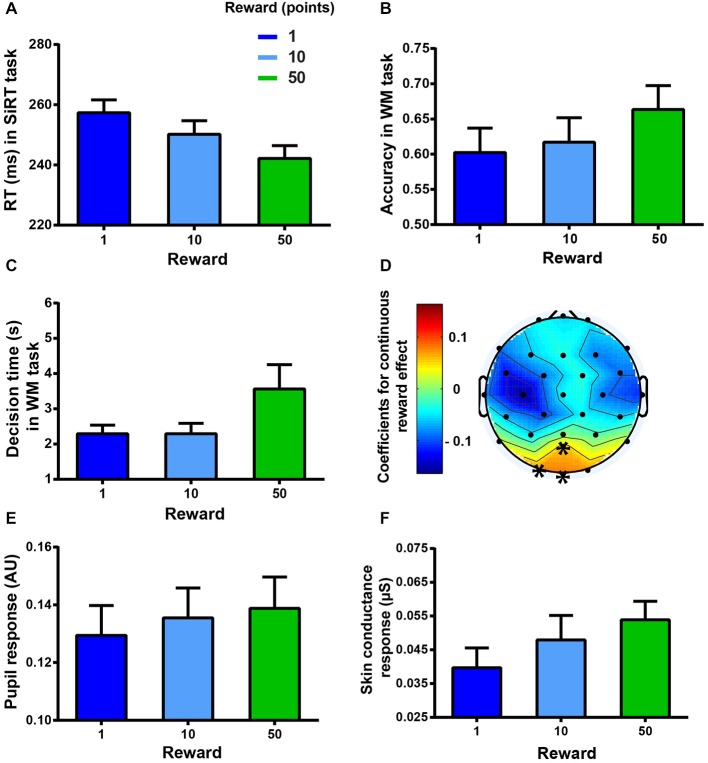
**Markers of motivation.** Error bars represent standard errors of the mean. **(A)** Mean RT (ms) as a function of the reward condition in the SiRT task. **(B)** Mean accuracy as a function of the reward condition in the WM task. **(C)** Mean decision time (s) as a function of reward condition in the WM task. **(D)** Coefficients of the reward effect on the high gamma band (60–140 Hz) activity in the WM task. Higher incentives led to higher activation on POz, Oz, and O1 electrodes (marked by asterisks, *p* < 0.05). **(E)** Mean pupil response as a function of reward condition in the WM task. **(F)** Mean SCR as a function of reward condition in the WM task.

A significant main effect of REWARD on the log-transformed RT gathered during the SiRT task showed that the participants responded faster for higher incentives (two-way RM ANOVA: *F*_(2,34)_ = 14.34, *p* < 0.0001, see Figure 4A). For this reward effect, *post hoc* tests with Bonferroni correction revealed that the subjects were significantly faster in the highest-reward condition (50 points) when compared to the medium (10 points) and to the lowest-reward conditions (1 point). Subjects were also significantly faster in the medium-reward condition than in the lowest-reward condition (all *p* < 0.005).

A significant main effect of REWARD was also found for the accuracy in the WM task (two-way RM ANOVA: *F*_(2,34)_ = 7.43, *p* < 0.001, see Figure 4B) showing that the subjects were more accurate when the incentive was the highest (Bonferroni corrected *post hoc* tests: 50 vs. 10 or 1, *p* < 0.05).

REWARD also affected the “decision time” between the display of the reward condition and the task onset, triggered by the subject through a key press (two-way RM ANOVA: *F*_(2,34)_ = 13.94, *p* < 0.0001, see Figure 4C). Indeed, subjects waited longer before starting the task when the reward was the highest (*post hoc* tests: 50-points vs. 10-points or 1-point reward conditions, *p* < 0.01).

In the WM task, some physiological variables were found to change in proportion to the reward incentive. High gamma band activity correlated significantly (*p* values following cluster-based correction < 0.05, see Figure 4D) with the reward incentive in the occipital region. Higher activity was observed with higher incentives on POz, O1 and Oz electrodes. The reward also had a significant effect on the pupil response (two-way RM ANOVA: significant main effect, *F*_(2,30)_ = 5.72, *p* < 0.01, see Figure 4E). Indeed, higher incentives led to larger pupil responses (*post hoc* tests: reward conditions 50 or 10 vs. reward condition 1, and 50 vs. 10, all *p* < 0.05). In addition a main effect of REWARD on the low-frequency responses of the SCR was found (two-way RM ANOVA: *F*_(2,18)_ = 5.8, *p* = 0.05, see Figure 4F), indicating that SCR increased as a function of higher incentives (*post hoc* tests: 50-points vs. 1-point reward condition, *p* < 0.05) while the other SCR frequency band (>0.1 Hz) did not show any significant effect (all *p* > 0.05).

Reward-induced changes in EEG and pupil response were found to correlate significantly with the reward-induced behavioral changes (ANCOVAs, EEG: *F*_(1,463)_ = 4.58, *p* = 0.0328; pupil response: *F*_(1,223)_ = 31.53, *p* < 0.0001), confirming that these measures can be regarded as markers of motivation. The effect of reward on SCR failed to correlate with the reward effect on behavior (ANCOVA: *F*_(1,132)_ = 1.07, *p* = 0.3037). This lack of correlation might be due to the smaller sample size obtained for the SCR analysis (*n* = 10); therefore, in this particular case, we analyzed the correlation with participant’s behavior slightly differently. We correlated the slope of the regression line obtained, for each subject, between reward and accuracy, on the one hand, and between reward and SCR, on the other hand. We found a significant positive relation between the reward effect on accuracy and behavior (Spearman correlation: *r* = 0.73, *p* = 0.0212) indicating that SCR can also be regarded as a marker of motivation.

### Evidence for Motivational Fluctuations During the Experiment

Regarding the subjective assessments of motivation, we found that the subjects’ task interest/enjoyment and perceived competence were significantly decreased at the end of the experiment (paired sample *t*-tests, interest/enjoyment: *t*_(17)_ = 6.30, *p* < 0.0001; perceived competence: *t*_(17)_ = 2.57, *p* < 0.05), while their effort and its perceived importance in task performance were not (paired sample *t*-test, effort/importance: *t*_(17)_ = −0.49, *p* = 0.625).

**Figure 5:**
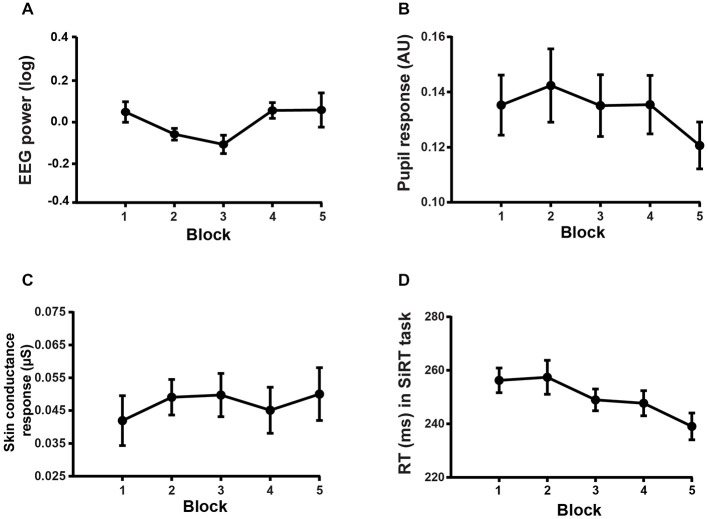
**Block effect on the markers of motivation. (A)** Mean EEG power averaged over the frequencies and electrodes isolated by the cluster-based permutation test in the WM task. **(B)** Mean pupil response as a function of block condition in the WM task. **(C)** Mean SCR as a function of block condition in the WM task. **(D)** Mean RT (ms) as a function of block condition in the SiRT task.

Conversely, two-way repeated measures ANOVAs on the physiological markers of motivation gathered in the WM task did not reveal any significant decrease across block repetition (EEG:* F*_(4,60)_ = 1.83, *p* = 0.1343, see Figure 5A; pupil response: *F*_(4,60)_ = 1.08, *p* = 0.37, see Figure 5B; SCR: *F*_(4,36)_ = 1.1, *p* = 0.37, see Figure 5C). The probability of this negative result being genuine was assessed by means of the Bayes factor (BF) estimation method (Masson, 2011). The BF indicates how much more likely the null hypothesis is with respect to the alternative hypothesis, given the data. For the abovementioned non-significant block effect on EEG, pupil response and SCR, the BF was equal to 102.3691, 443.0648 and 158.2374, respectively. These BFs provide strong evidence in favor of the null hypothesis.

We found a significant main effect of BLOCK on RT in the SiRT task (two-way RM: *F*_(4,68)_ = 12.31, *p* < 0.0001, see Figure 5D). Multiple comparisons with Bonferroni correction showed that the participants were significantly faster (all *p* < 0.05) in the 3rd, 4th and 5th blocks than in the 1st block. Furthermore, a significantly faster RT was found in the last block than in the 2nd and 3rd block (all *p* < 0.05). This finding that responses get faster across block repetition in the SiRT task argues also against a decrease in motivation over time.

### Interaction Between MF and Motivation

**Figure 6:**
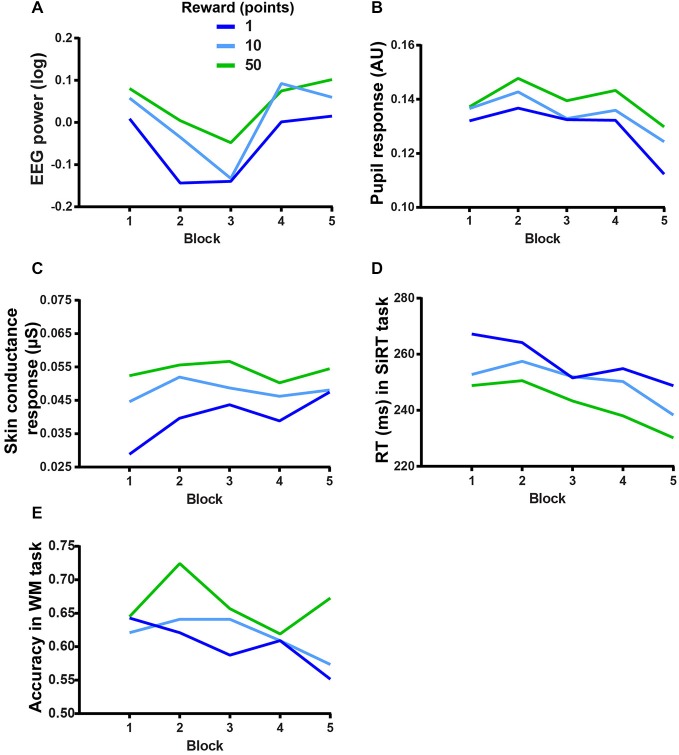
**Reward-block interactions on (A) EEG power during the WM task (averaged over the cluster), (B) pupil response during the WM task, (C) SCR during the WM task, (D) RT in the SiRT task and (E) accuracy in the WM task**.

Finally, in order to evaluate whether the extrinsic motivational manipulations interacted with the fatigue effect, we looked at the interactions between REWARD and BLOCK. The two-way repeated measures ANOVAs on physiological markers of motivation failed to show any significant interaction between REWARD and BLOCK (EEG: *F*_(8,120)_ = 0.52, *p* > 0.5, BF = 5.49 × 10^7^, see Figure 6A; pupil response: *F*_(8,120)_ = 0.68, *p* > 0.5, BF = 1.7 × 10^7^, see Figure 6B; SCR: *F*_(8,72)_ = 0.78, *p* > 0.5, BF = 1119900, see Figure 6C) indicating that the motivational value of the reward did not change throughout the experiment. This lack of interaction was also found on performance in the SiRT (*F*_(8,136)_ = 0.74, *p* = 0.65, BF = 1.81 × 10^8^, see Figure 6D).

Regarding the subjective assessment of motivation, the decrease in the interest/enjoyment subscale of the IMI did not significantly correlate with either the subjective (general fatigue subscale of the MFI) or the objective measure of MF (objective fatigue index, see “Evidence for MF Emergence During the Experiment” Section). The non-significant correlation between motivation and either the subjective (Pearson correlation: *r* = 0.31, *p* = 0.21, BF = 1.6973) or objective measure of fatigue (Pearson correlation: *r* = −0.15, *p* = 0.55, BF = 3.4664) indicated that the increase in the subjective feeling of fatigue and the drop in performance cannot be attributed to a decrease in intrinsic motivation. Correlation between the perceived competence subscale of the IMI and the subjective or objective measures of fatigue did not also reveal any significant relation (Spearman correlation: all *p* > 0.1).

According to our third prediction, the performance in the WM task should have dropped more in the low stake condition than in the high stake condition. However, the interaction between REWARD and BLOCK on the performance in the WM task accuracy was not significant (*F*_(8,136)_ = 1.28, *p* = 0.2508, BF = 3.02 × 10^8^, see Figure 6E), indicating that the detrimental effect of MF (performance decrement) could not be overcome even when the incentives were high (50 points).

## Discussion

In the present study, we attempted to find evidence for the causal role of a progressive decrease of task-related motivation in the emergence of MF. We evaluated MF by means of subjective ratings, behavioral performance and physiological markers. Despite a very significant increase in subjective fatigue at the end of the experiment, the deterioration of behavioral performance in the WM task was small. This is however a common finding in the literature (DeLuca, 2005), possibly because subjects maintain their performance despite MF by means of a compensatory increase in mental effort (Hockey, 1997; Nakagawa et al., 2013; Esposito et al., 2014). In addition, we found that two well-known psychophysiological signatures of MF, namely long-term HRV (SD2) (Mukherjee et al., 2011) and blink rate (Stern et al., 1994), increased over time and correlated with the concurrent drop in performance. In contrast, the drop in heart rate, known for long to occur concurrently to the development of fatigue (Arai, 1912), failed to correlate with the fatigue indices; to the best of our knowledge, it is the first time that correlations between these measures are investigated. It could thus be proposed that the drop in heart rate corresponds in fact to a phenomenon that, although occurring concomitantly with fatigue, bears no relation to the mechanisms involved in the development of MF, such as the adoption of a prolonged sedentary position.

Along the same lines, it could also be argued that the decrease in performance and the changes in the physiological markers of MF could have been caused by other phenomena, such as a drop of vigilance (Oken et al., 2006). Changes in the level of vigilance are defined operationally as a slowing of the RTs in easy detection tasks after a prolonged period of time (Pattyn et al., 2008). Here, on the contrary, we observed a clear decrease of RT over time in the SiRT task, indicating that there was no drop of vigilance over the course of our experiment. We also failed to find the classical EEG signature of vigilance drops, consisting in an increase in the theta frequency band over time (Paus et al., 1997). Along the same line, the eye blink parameters that are regarded as markers of drowsiness and drops in vigilance i.e., the average blink duration and the proportion of long blinks (Caffier et al., 2003; McIntire et al., 2014), failed to change with time-on-task, also arguing against changes in drowsiness/vigilance over time in our experiment. Thus it seems sensible to conclude that behavioral and physiological block-related changes that we observed in the current study were indeed specific markers of progressive MF.

The hypothesis that MF would be caused by a loss of motivation led to a series of testable predictions. First, we expected the indicators of motivation to decrease across block repetition. To tackle this prediction, we measured motivation by means of subjective ratings, behavioral performance and physiological markers. We were able to isolate three physiological indicators of motivation that increased with reward and correlated with its behavioral effects, namely high gamma band activity in the occipital EEG, pupil size, and SCR. The pupil and SCR responses to motivation confirmed the conclusion of earlier studies reporting a relationship between these physiological variables and motivational status (Kahneman and Peavler, 1969; Schmidt et al., 2008). However, the EEG correlate of motivation was less expected. To our knowledge, it is the first time that high gamma power increases in the occipital region are described in relation to motivation, which is classically associated with power suppression in the alpha frequency range (Keil et al., 2006; Ewing and Fairclough, 2010). However, a recent study (Ossandón et al., 2011) has described similar electrophysiological phenomena in relation to manipulations of the task difficulty. A study using fMRI also revealed an enhanced activation in the visual association cortex as a function of higher incentive during the performance of a WM task (Krawczyk et al., 2007) and other studies have also described increased gamma band activity in other parts of the brain in relation to motivation or task engagement (Fründ et al., 2007; Mulert et al., 2007; Tan et al., 2013; Bosman et al., 2014). It could be hypothesized that an increase in mental effort, which can be caused both by higher task difficulty and higher motivation (Kurzban et al., 2013), would be responsible for the changes in the occipital region that we observed. Finally, it is worth mentioning that these reward-related EEG changes could not be elicited by the visual features of the display used to inform the subjects about the reward condition, since the EEG signals included in the analyses followed the offset of this display.

Amongst the motivational markers we evaluated, only the subjective ratings indicated a decrease over time, while the other indicators failed to show any significant change. The difference between the subjective evaluation and objective measures of motivation might result from the fact that the interest/enjoyment subscale of the IMI evaluates the pleasure experienced during the task execution and the perceived competence subscale estimates how the participants perceive their performance in the task while the objective motivational measures could relate more to the motivation-dependent mental effort invested during the task (Heitz et al., 2008; Venables and Fairclough, 2009). In accordance with this view, we found that the effort/importance subscale of the intrinsic motivation questionnaire failed to show any change at the end of the experiment. Therefore, it appears that while the subjects maintained their mental effort throughout the experiment, their subjective experience of the task changed, becoming more and more unpleasant over time.

Our second prediction was that the behavioral decrease in performance over time should be proportional to the progressive build-up of fatigue. All the correlation analyses showed that the blockwise changes in motivation, evaluated by the different measures described above, did not vary commensurately with the progressive increase in MF. This provides strong evidence that any motivational changes that might occur during the experiment cannot be causally linked to the concurrent development of MF.

Third, we predicted that reward manipulations, by compensating a possible decrease of motivation, would, at least partly, alleviate the effects of fatigue. In contrast, the absence of reward-block interaction in the different markers of motivation indicates that reward manipulations failed to compensate the effect of MF. Indeed, if fatigue were caused by a loss of motivation, we would have expected the performance to drop more in the low-reward condition than in the high-reward condition, thereby resulting in a significant interaction between reward and block order. However, our results show that the subjects could not overcome the detrimental effects of MF, despite a high extrinsic motivation. This lack of effect cannot be explained by a progressive loss of interest in the monetary incentive, since the effect of reward on the SiRT task performance and on all the motivational indicators described above remained unchanged during the course of the experiment.

Finally, given that the SiRT task was influenced by motivational manipulations, we expected MF to affect performance during this task as well. However, we found that the performance in the SiRT task increased over time, clearly indicating that it was not affected by MF.

Since the present findings consist mostly in absence of effects, we attempted to evaluate how strongly we can trust these negative results on the basis of BF analyses (Masson, 2011). Most of these analyses provided strong to very strong evidence in favor of the null hypothesis. However, the correlations between the subjective motivational variables and the subjective fatigue index provided only weak evidence. Thus, we cannot affirm with certainty that larger samples would not allow to uncover a significant relation between some of these variables. However, given the caveats discussed above regarding the interpretation of the subjective intrinsic motivation variables, and given that all the other analyses provided very clear evidence against the main hypothesis of a causal link between fatigue and motivational loss, we do not believe that this slightly less conclusive finding should significantly alter our conclusion.

A noteworthy limitation of the present study, which is in fact common to many fatigue-related studies, is the lack of a control condition in which no fatigue would be induced. The decision not to include such a control condition was made because of the difficulty of choosing an appropriate control task—which had to be neutral in terms of fatigue and motivational effects—and because it was possible to circumvent this shortcoming by relying on correlation analyses between the fatigue indices and the changes observed in the dependent variables. This method allowed us to maintain a reasonable level of specificity of the fatigue effects, despite the absence of control. Future studies, using such a control condition, could provide a more robust confirmation of the present findings.

To sum up, the present results indicate that MF is not caused by a progressive disengagement from the task, or motivational decline. Importantly, we do not claim that drops in motivation never happen concurrently to MF, nor that these motivational drops never contribute to MF but rather that loss of motivation cannot be considered as a necessary causal factor in the development of MF. These findings strongly question the mechanisms actually responsible for MF. These likely engage either the disruption of cognitive resources—for instance through progressive metabolic alterations—or changes in cognitive control impeding the availability of these resources (van der Linden et al., 2003; Hockey, 2011; Kurzban et al., 2013; Ishii et al., 2014). Future work should aim at addressing the nature of these metabolic and/or functional alterations.

## Author Contributions

AZ designed the experiments with the support of all other authors. The scripts necessary to run the tasks used in the experiments (Sudoku task, WM task and SiRT task) were programmed by BJ. The data were acquired by MG and analyzed by AZ and MG. All authors contributed to the writing of the manuscript.

## Conflict of Interest Statement

The authors declare that the research was conducted in the absence of any commercial or financial relationships that could be construed as a potential conflict of interest.

## Footnotes

^1^http://www.sudokuoftheday.co.uk/

^2^http://www.selfdeterminationtheory.org/intrinsic-motivation-inventory/

^3^http://nocions.webnode.com/letswave/

## Abbreviations

MF: mental fatigue
WM: Working memory
SiRT: simple RT task
MFI: Multidimensional Fatigue Inventory
IMI: Intrinsic Motivation Inventory.
